# Letters to the Editor

**DOI:** 10.1155/S1064744901000114

**Published:** 2001

**Authors:** Elliott M. Levine

**Affiliations:** Illinois Masonic Medical Center Chicago IL USA


### Letters to the Editor

**To the Editor:**

I found it very interesting to read 'Liberal diagnosis and treatment of intrauterine infection reduces early-onset neonatal group B streptococcal infection but not sepsis by other pathogens' by H. Wolf and colleagues *(Infect Dis Obstet Gynecol* 2000;8:143–50). My coauthors and I arrived at the same conclusion from our investigations on this matter. We also found that 'the overall incidence of neonatal sepsis remained unchanged following' the 'increased use of intrapartum chemoprophylaxis', in spite of the reduction in GBS neonatal sepsis[1]. Although we were encouraged to see others confirm our reported findings, we were disappointed in that our paper was not referenced, even though it was published earlier in the same journal as this one by Wolf and colleagues.

*Elliott M. Levine, MD*
*Illinois Masonic Medical Center*
*Chicago, IL*

1. Levine EM, Ghai V, Barton JJ, Strom CM. Intrapartum antibiotic prophylaxis increases the incidence of Gram-negative neonatal sepsis. *Infect Dis Obstet Gynecol* 1999;7:210–13

### Author's response

Although we concluded that evaluation of preventive measures for early-onset group B streptococcus (GBS) sepsis should always take the incidence of neonatal sepsis caused by other pathogens into account, we unfortunately overlooked one of the few studies that did so. Because of the retrospective design with historical comparison of the study of Levine and colleagues and of our study, factors other than the increase in antibiotic administration could be responsible for the finding that the incidence of neonatal GBS sepsis was reduced while the overall incidence of neonatal sepsis did not change. Confirmation by others is therefore important, as Levine and colleagues rightly concluded.

Interestingly, Levine and colleagues observed an increase of neonatal sepsis caused by *Escherichia coli,* whereas in our study sepsis caused by *Staphylococcus* species increased but *E. coli* did not. Possibly this difference is caused by the use of ampicillin by Levine and colleagues and amoxicillin with gentamicin, which is more effective against gram-negative pathogens, in our study. It is presently not known whether prophylaxis with penicillin instead of ampicillin, as advised by the Centers for Disease Control report in 1996[1], causes a change in resistance pattern and an increase in sepsis caused by other pathogens. We are not aware of any published studies in this regard.

*H. Wolf[1], A.H.P. Schaap[1], B.J. Smit[2], L. Spanjaard[3] and A.H. Adriaanse[1]*
*[1]Departments of Obstetrics and Gynecology,*
*[2]Neonatology and [3]Microbiology of the Academic Medical Center,*
*PO Box 22660, 1100 DD Amsterdam,*
*The Netherlands*

1. Anonymous. Prevention of perinatal group B streptococcal disease: a public health perspective. Centers for Disease Control and Prevention. *Morb Mortal Wkly Rep* 1996;45:1–24
